# Insecticide resistance status and mechanisms in *Aedes aegypti* and *Aedes albopictus* from different dengue endemic regions of Panama

**DOI:** 10.1186/s41182-024-00637-w

**Published:** 2024-10-09

**Authors:** Lorenzo Cáceres Carrera, Luis Piedra, Rolando Torres-Cosme, Anakena M. Castillo, Antonio Bruno, José Luis Ramírez, Dan Martínez, María Magdalena Rodríguez, Juan A. Bisset

**Affiliations:** 1 Departamento de Entomología Médica del Instituto Conmemorativo Gorgas de Estudios de la Salud, PO. Box 0816-02593, Panamá, Panamá; 2 Deparatamento de Control de Vectores del Instituto de Medicina Tropical “Pedro Kourí”, La Habana, Cuba; 3 Departamento de Química de Alimentos y Aguas del Laboratorio Central de Referencia en Salud Pública del Instituto Conmemorativo Gorgas de Estudios de la Salud, Ciudad de Panamá, Panamá; 4 Agricultural Research Service, United States Department of Agriculture. Crop Bioprotection Research Unit, National Center for Agricultural Utilization Research, Peoria, IL USA

**Keywords:** *Ae. Aegypti*, *Ae. Albopictus*, Insecticide, Resistance, Susceptibility, *Kdr* mutation

## Abstract

**Background:**

Dengue is a serious public health problem worldwide, including Panama. During the last years, the number of dengue cases has increased. This may be due to the presence of mosquito populations resistant to insecticides. The aim of this study was to characterize the resistance status, its enzymatic mechanisms and *Kdr* mutations in wild populations of *Aedes aegypti* and *Aedes albopictus*.

**Methods:**

Standard WHO bioassays were performed using insecticide-treated filter papers to determine resistance in populations *Ae. aegypti* and *Ae. albopictus* to pyrethroids insecticides, organophosphates, to the carbamate propoxur and to the organochlorine DDT. Biochemical assays were conducted to detect metabolic resistance mechanisms and real-time PCR was performed to determine the frequencies of the *Kdr* mutations Val1016IIe and F1534C.

**Results:**

The strains *Ae. aegypti* El Coco showed confirmed resistance to deltamethrin (78.5% mortality) and lambda-cyhalothrin (81%), Aguadulce to deltamethrin (79.3%), David to deltamethrin (74.8%) and lambda-cyhalothrin (87.5%) and Puerto Armuelles to permethrin (83%). *Aedes aegypti* El Empalme showed confirmed resistance to pirimiphos-methyl (62.3% mortality), chlorpyrifos-methyl (55.5%) and propoxur (85.3%). All strains of *Ae. albopictus* showed possible resistance to PYs and five strains to DDT. Only *Ae. albopictus* Canto del Llano showed confirmed resistance to pirimiphos-methyl (70% mortality) and malathion (62%). Esterase activity was variable across sites with the most frequent expression of α-EST compared to β-EST in *Ae. aegypti* populations. In *Ae. Albopictus*, the expressed enzymes were β-EST and MFOs. Through ANOVA, significant differences were established in the levels of enzymatic activity of α- and β-EST, MFOs and GST, with *p* < 0.001 in the *Ae. aegypti* and *Ae. albopictus.* The *Kdr* Val1016IIe mutation was detected in *Ae. aegypti* Aguadulce, El Coco and David. The odds ratio for the Val1016Ile mutation ranged from 0.8 to 20.8 in resistant mosquitoes, indicating the association between pyrethroid phenotypic resistance and the kdr mutation.

**Conclusion:**

The presence of a varied and generalized resistance, enzymatic mechanisms and the Val1016IIe mutation may be associated with the intensive use and possibly misuse of the different insecticides applied to control *Aedes* populations. These results highlight the need to develop a program for resistance management. Also, alternative approaches to mosquito control that do not involve insecticides should be explored.

## Introduction

Dengue is a mosquito-borne disease that has the greatest epidemic potential in the world. The *Ae. aegypti* mosquito is the main vector of dengue in the Americas [1]. However, in a study carried out on the transmissibility potential, the results showed that *Ae. aegypti* presented higher infection levels than *Ae. albopictus*, indicating that *Ae. albopictus* could be contributing to the spread of the dengue and chikungunya virus in large areas of America and Europe [2]. The Americas is one of the most affected regions [3], where dengue is considered one of the most important reemerging diseases [4]. The risk scenario of mosquito-borne diseases has changed dramatically in the last decades due to the emergence and re-emergence of urban transmission cycles caused by *Ae. aegypti* and *Ae. albopictus* [5]*.* The current situation against dengue (DENV), Zika (ZIKV) and chikungunya (CHIKV) arboviruses transmitted by *Ae. aegypti* and *Ae. albopictus* is complex because so far there is no vaccine with high effectiveness and no specific treatment, except the vaccine against yellow fever. Therefore, mosquito vector control is the only solution to prevent these diseases. However, this remains a challenge despite the existence of vector control programs that have been in place for several decades [6].

Historically, the main strategies used for the control of arbovirus vector mosquito populations, specifically *Ae. aegypti* [7] rely heavily on the use of insecticides widely applied by vector control programs [8]. In recent years, resistance to the four major chemical groups of organochlorines (OC), organophosphates (OPs), carbamates (CA) and pyrethroids (PYs) insecticides has been detected in *Ae. albopictus* in the Americas, Africa and Asia [9–11]. Insecticide resistance in mosquitoes is caused by several mechanisms, with two in particular being the focus of most studies: metabolic resistance and target-site alterations modifications. Metabolic resistance involves large families of enzymes: cytochrome P450 monooxygenases (MFO), esterases (EST), glutathione *S*-transferases (GST) and carboxylesterases (CCE). Moreover, increased activity levels of insecticide-degrading enzymes have been observed in resistant populations [12, 13].

Studies have suggested that mutations in the voltage-dependent sodium channel (NaV), the target site for PYs and OC, may play a role in PYs resistance [14]. To date, at least 11 NaV detected mutations associated with resistance in *Ae. aegypti* [15]. Four of these, S989P, I1011M, V1016G and F1534C, have been functionally confirmed to confer resistance to PYs insecticides [12, 16]. The most common alleles in the Americas are 410L + 1016I + 1534C, 410L + 1534C and 1534C [17]. The mutations G923W, L982W, I1011M and V1016G were found in permethrin- and DDT-resistant *Ae. aegypti* populations from Asia and Brazil [18], while substitutions I1011V and V1016I were found in *Ae. aegypti* populations from Latin America [19]. In addition, the F1534C was discovered recently in Brazil [20], Venezuela [21] and Colombia [22]. In the Americas, the Val1016Ile mutation was found to coexist with F1534C in Venezuela [21] and Brazil [23].

In Panama, the main tool used by the vector control program against mosquito populations is the use of chemical insecticides. *Aedes* populations have been controlled since the beginning with the organochlorine DDT, later with OPs temephos, fenthion, malathion and fenitrothion, and more recently, the PYs insecticides deltamethrin and cyfluthrin [24, 25]. *Aedes aegypti* has shown resistance to the insecticides OPs temephos, pirimiphos-methyl and chlorpyrifos-methyl, the PYs insecticides deltamethrin, cyfluthrin and cypermethrin and to the organochlorine DDT. In more recent studies in bioassays with adult mosquitoes, resistance was recorded in two populations of *Ae. aegypti* to pirimiphos-methyl, fenitrothion, malathion and propoxur. *Aedes* populations were completely susceptible to pyrethroids [26–28]. Through biochemical assays, EST, MFOs and GST were observed as mechanisms of resistance to OPs insecticides [27, 28]. Regarding *Kdr* (Knockdown resistance) mutations, the mutations Ile1011Met and Val1016Gly were recently detected in a population of *Ae. aegypti* [29]*.*

Despite the continuous use of insecticides against *Aedes* populations, few studies have been conducted on the resistance status in *Ae. aegypti* and *Ae. albopictus* populations in the different regions of the country, especially on the enzymatic mechanisms of resistance and *Kdr* mutations*.* The National *Aedes* Control Program (PNCA) of the Ministry of Health (MINSA) has continuously expressed the need to conduct studies to determine the resistance status in *Aedes* populations. The objective of this study was to evaluate the status of insecticide resistance, its enzymatic mechanisms and *Kdr* mutations in wild *Ae. aegypti* and *Ae. albopictus* populations from Panama.

## Materials and methods

### Study sites

**Fig. 1:**
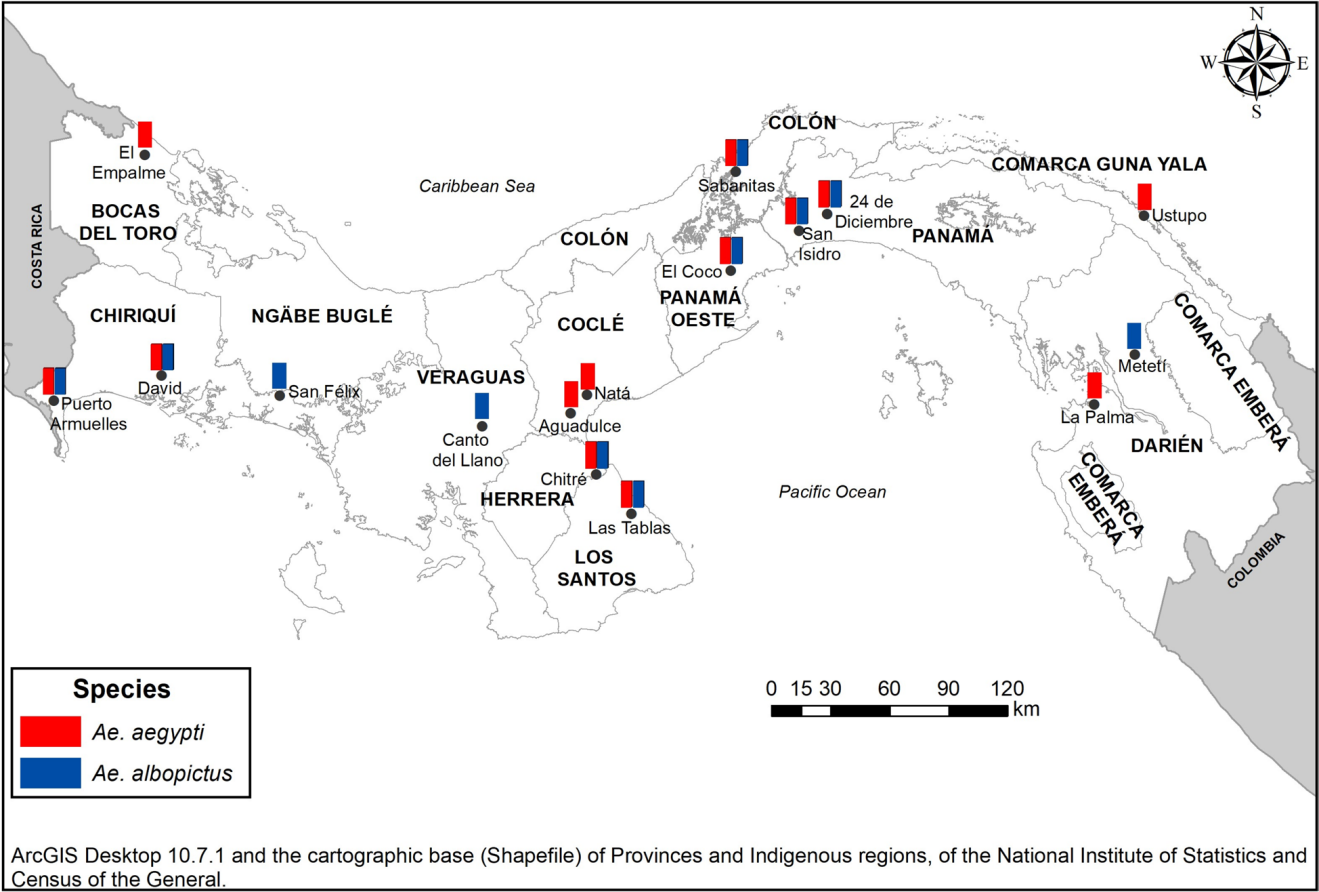
Geographic location map of the sites studied and species of *Aedes* collected

The PNCA raised the need to determine the resistance status of applied and alternative insecticides against *Ae. aegypti* and *Ae. albopictus* in sites of epidemiological importance considering the number of reported cases of dengue, and entomologically due to the high infestation rates recorded and the frequent applications of insecticides. In conjunction with the PNCA, a total of 16 communities (urban and semi-urban) were selected, located in 16 municipalities of Panama. Table 1 shows the geographic coordinates of the selected sites and epidemiological data on reported dengue cases and Fig. 1 presents the map with the geographical location of the studied sites and mosquito species collected.

**Table 1:** Geographical and epidemiological data of dengue cases reported in the sites where *Ae. aegypti* and *Ae. albopictus* strains were collected to evaluate their insecticide resistance status. Panama, 2016–2022

Province Comarca*	Municipality	Locality	Coordinates	Altitude (meter)	DENV Accumulated Cases 2016 to 2020**
Bocas del Toro	Changuinola	El Empalme	9° 24′ 42.53ʺ N	15 m	1936
			82° 31′ 16.88ʺ O		
Coclé	Aguadulce	Aguadulce	8° 14′ 01.40ʺ N	22 m	1660
			80° 32′ 11.87ʺ O		
	Nata	Natá	8° 20′ 14.08ʺ N	14 m	
			80° 31′ 06.02ʺ O		
Colón	Colón	Sabanitas	9° 21′ 04.14ʺ N	8 m	2743
			79° 47′ 58.34ʺ O		
Chiriquí	David	David	8° 22′ 59.76ʺ N	20 m	562
			82° 25′ 34.37ʺ O		
	Barú	Puerto Armuelles	8° 15′ 37.91ʺ N	15 m	
			82° 52′ 03.38ʺ O		
Darién	Chepigana	La Palma	8° 24′ 19.16ʺ N	42 m	572
			78° 08′ 24.09ʺ O		
	Pinogana	Meteti	8° 30′ 19.16ʺ N	48 m	
			77° 58′ 15.68ʺ O		
Herrera	Chitre	Chitre	7° 58′ 04.71ʺ N	34 m	1172
			80° 26′ 11.01ʺ O		
Los Santos	Las Tablas	Las Tablas	7° 45′ 57.98ʺ N	44 m	837
			80° 16′ 35.04ʺ O		
Guna Yala*	Guna Yala	Ustupo	9° 07′ 50.31ʺ N	5 m	300
			77° 55′ 34.59ʺ O		
Ngäbe Buglé*	San Félix	San Félix	8° 17′ 34.73ʺ N	112 m	13
			81° 51′ 56.49ʺ O		
Panamá	Panamá	24 de Diciembre	9° 05′ 56.63ʺ N	23 m	10,572
			79° 21′ 53.84ʺ O		
	San Miguelito	San Isidro	9° 04′ 02.71ʺ N	98 m	
			79° 30′ 29.87ʺ O		
Panama Oeste	La Chorrera	El Coco	8° 52′ 13.74ʺ N	82 M	2319
			79° 47′ 56.59ʺ O		
Veraguas	Santiago	Canto del Llano	8° 06′ 42.63ʺ N	94 M	570
			80° 57′ 46.93ʺ O		

*Comarca: The term “comarca” refers to the political division or territory assigned to a defined indigenous population within Panama

**: Source MINSA

### Collection and mosquito rearing

Collections of *Ae. aegypti* and *Ae. albopictus* in immature stages (larvae and pupae) were carried out in the intra- and peridomicile in different ecological environments located in urban and semi-urban areas. All biological materials collected were placed in special containers previously coded and transported to the Department of Medical Entomology (DME) of the Instituto Conmemorativo Gorgas de Estudios de la Salud (ICGES) for identification to species level using taxonomic keys of mosquito larvae and pupae [30]. The biological material of adult females produced from larvae and pupae of *Ae. aegypti* and *Ae. albopictus* collected in the field was called the F_0_ generation. The first generation (F_1_) of the different strains of *Ae. aegypti* and *Ae. albopictus* was generated from the postures produced from the F_0_ and used to raise colonies in laboratory conditions, with average temperature of 28.5 °C to 30 °C and relative humidity of 70–80% and with a photoperiod of 12:12 (day/night).

### Susceptibility bioassays with adult mosquitoes

Resistance profiling of the different strains of *Ae. aegypti* and *Ae. albopictus* was performed using bioassays with papers impregnated with the organochlorine DDT (4%), the PYs deltamethrin (0.03%), lambda-cyhalothrin (0.03%), cyfluthrin (0. 15%) and permethrin (0.25%), the OPs fenitrothion (1%), malathion (5%), chlorpyrifos-methyl (0.4%) and pirimiphos-methyl (0.25%) and the CA propoxur (0.1%) with the diagnostic doses and exposure times established in the WHO standardized protocol [31]. Each test with the insecticides evaluated had three replicates and their respective controls. After the exposure period, the mosquitoes were transferred to the recovery chambers and a cotton moistened in 10% sucrose solution was placed as food during the recovery period. The reference strains *Ae. aegypti* Rockefeller (Rock) and *Ae. albopictus* Fraga were used as the susceptible standard (Fraga: *Ae. albopictus* susceptible strain it has been kept in the insectary of the Institute of Tropical Medicine Pedro Kouri, La Habana, Cuba, since 2012). The percent mortality in the exposure and control tubes was recorded at 24 h.

### Biochemical assays

Thirty individual larvae from each field colony were assayed for α and β-EST [32], MFOs [33], GST [34] and AchE [35]. Each fourth-instar larva was homogenized in 300 μL of 0.01 M sodium phosphate buffer (pH 7.5), and 20 μL of the crude homogenate was transferred separately to a microtiter plate for each enzymatic assay, and two replicates of 10 μL were added to another plate for protein assay. Absorbance levels were measured spectrophotometrically on a TECAN Sunrise Basic Microplate Reader (TECAN Austria GmbH 5082 Grodig, Austria), at wavelengths indicated for each enzyme, and the mean absorbance per larvae calculated based on data for the two replicate wells.

### Detection of Val1016Ile and F1534C sodium channel mutations

The mosquito strains were exposed to deltamethrin and 30 surviving individuals F1 from each strain were taken to detect both sodium channel gene mutations at positions 1016 and 1534 from genomic DNA by allele-specific PCR (AS-PCR). Primer sequences for both mutations are shown in Table 2 [19, 36]. The total DNA from 30 *Ae. aegypti* mosquitoes was extracted using the Livak method [37, 38].

**Table 2:** Sequence of primers for detection of *Kdr* mutations (Val1016Ile and F1534C) in *Ae. aegypti* populations

Phenotype	Primers name	Primer sequence (5ʹ → 3ʹ)	bp
V10I6I^a^	V1016f	[GCGGGCAGGGCGGCGGGGGCGGGGCC]ACAAATTGTTTCCCACCCGCAC*C*G**G**	51
	I1016f	[GCGGGC]ACAAATTGTTTCCCACCCGCA*CT*G**A**	31
	Iso1011r^c^	TGATGAACCSGAATTGGACAAAAGC	25
F1534C^b^	AaEx31P	5ʹTCGCGGGAGGTAAGTTATTG3ʹ	19
	AaEx31Q	5ʹGTTGATGTGCGATGGAAATG3ʹ	20
	AaEx31wt	5ʹCCTCTACTTTGTGTTCTTCATCATCTT3	27
	AAEX31MUT	5ʹGCGTGAACGACCCGC3	18

This sequence primers is associated with resistance to pyrethroid insecticides

Primers feature base pair mismatches introduced at the third base from 3ʹ end to increase allele specificity (italics); the diagnostic differential nucleotide is in bold, underlined. For Val1016 to Ile (Iso) the diagnostic nucleotide is the first in the codon (GTA to ATA) while for F1534C is the second nucleotide of the codon (TTC or TTT to TGA or TGC)

^a^Saavedra-Rodriguez et al.[15]

^b^Yanola et al.[34]

^c^Pinto et al.[35]

The amplification of the 1016 site was performed following the protocol described by Saavedra-Rodriguez et al. [19], with the exception of using an improved common reverse primer from Pinto et al. [39]. Melting curve analyses for the F1534C were as reported by Yanola et al. [40]. The concentrations of the PCR reagents were calculated for a volume of 25 μL. Each reaction contained a final concentration of 5X Green Gotaq Flexi Buffer (Promega), 2.5 mM of MgCl_2_, 0.4 mM of each dNTPs, 0.5 μM of each primer, 2.5 U of GoTaq G2 Flexi DNA polymerase (Promega) and 2 μL of genomic DNA previously extracted from a mosquito as a mold and subjected to a thermocycler under the following conditions 95 °C for 5 min, followed by 35 cycles of 95 °C for 30 s, 63 °C for 1 min and 72 °C for 30 s and its final extension step 72 °C for 10 min. The amplification products were observed in an agarose gel, LMP, Analytical Grade (Promega) at 2% low melting point due to the small difference between the amplicons.

The PCR reaction for the detection of the F1534C allele was calculated for a volume of 25 μL following the method described by Harris et al. [36]. The final concentrations of the reagents contained 5X Gotaq Flexi Green PCR buffer (Promega), 2.5 mM of MgCl2, 0.4 mM of each dNTPs, 0.5 μM of each primer, 2.5 U of GoTaq G2 Flexi DNA polymerase (Promega) and 2 μL of genomic DNA previously extracted from a mosquito as a template and subjected to a thermocycler (T-100, Bio-Rad) under the following conditions 95 °C for 5 min followed by 35 cycles of 94 °C for 30 s, 63 °C for 30 s and 72 °C for 30 s, and a final extension step of 72 °C for 10 min. The PCR products were visualized on an agarose gel, LE, Analytical Grade (Promega) at 2%.

### Statistical analysis

Mortality rates recorded during bioassays were analyzed according to the WHO criteria. The populations of *Aedes* were classified as “confirmed resistance” if less than 90% mortality was observed, as “possible resistance” if mortality rates were between 90 and 98% and “susceptible” for more than a 98% mortality rate [31]. If the control mortality is ≥ 5% and < 20%, the mortality should be corrected by Abbott's formula [41]. In biochemical tests, total protein was measured for each mosquito using the method of Bradford [42]. Enzyme activity was classified as “unaltered” between 0 and 15%, “incipient altered” between 15 and 50% and “altered” between 50 and 100% [43]. Data for each biochemical assay were evaluated via analysis of variance (ANOVA). Differences were considered significant at *p* < 0.05. Tukey’s test was conducted after significant differences had been identified by ANOVA to establish which means differed from reference strains. The genotype frequencies were calculated by dividing the number of individuals with a given genotype (position of the bands in agarose gel electrophoresis) by the total number of analyzed mosquitoes as follows: Fg = Quantity AA/N (%). The chi-square test (*X*^2^) was used to determine the association between V1016I and F1534C sodium channel mutations and the resistance phenotype. This statistical test was carried out using EPIDAT version 3.1. AA: a. homozygous wild genotype frequency (V1016/V1016 or F1534/F1534), b. homozygous mutant genotype frequency (I1016/I1016 or 1534C/1534C), c. heterozygote genotype frequency, (V1016/I1016 or F1534/C1534).

## Results

### Susceptibility bioassays with adult mosquitoes

**Fig. 2:**
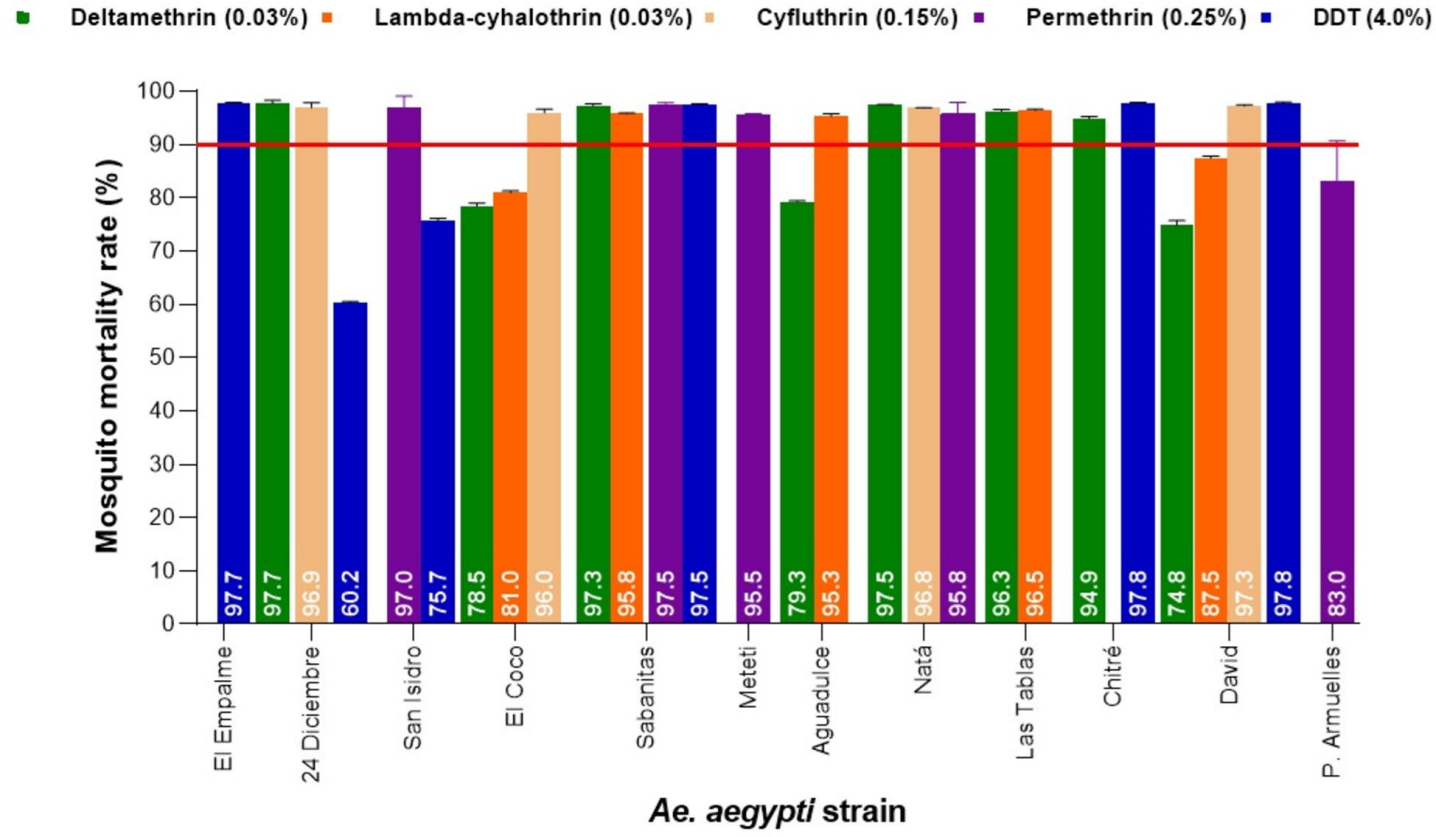
Resistance status in populations of *Ae. aegypti* adults evaluated by diagnostic concentrations of pyrethroid and organochlorine insecticides using WHO standardized bioassays. The continuous red line shows the threshold value of 90%, below this value a population is considered resistant to insecticides

Considering the resistance threshold according to the WHO protocol, it was found that a total of 12 strains of *Ae. aegypti* presented variable resistance to the insecticides PY and two to DDT. In bioassays, strains *Ae. aegypti* 24 Diciembre with 60.2% and San Isidro with 75.7% mortality showed confirmed resistance to DDT, *Ae. aegypti* El Coco to deltamethrin (78.5%) and lambda-cyhalothrin (81%), *Ae. aegypti* Aguadulce to deltamethrin (79.3%) and possible resistance to lambda-cyhalothrin (95.3%), *Ae. aegypti* David to deltamethrin (74.8%) and lambda-cyhalothrin (87.5%) and *Ae. aegypti* Puerto Armuelles to permethrin (83%). The rest of the strains had possible resistance to at least one insecticide. *Aedes aegypti* La Palma and *Ae. aegypti* Ustupo strains showed no resistance to any insecticide. Figure 2 shows the resistance status of the different *Ae. aegypti* populations to PYs insecticides and the organochlorine DDT.

**Fig. 3:**
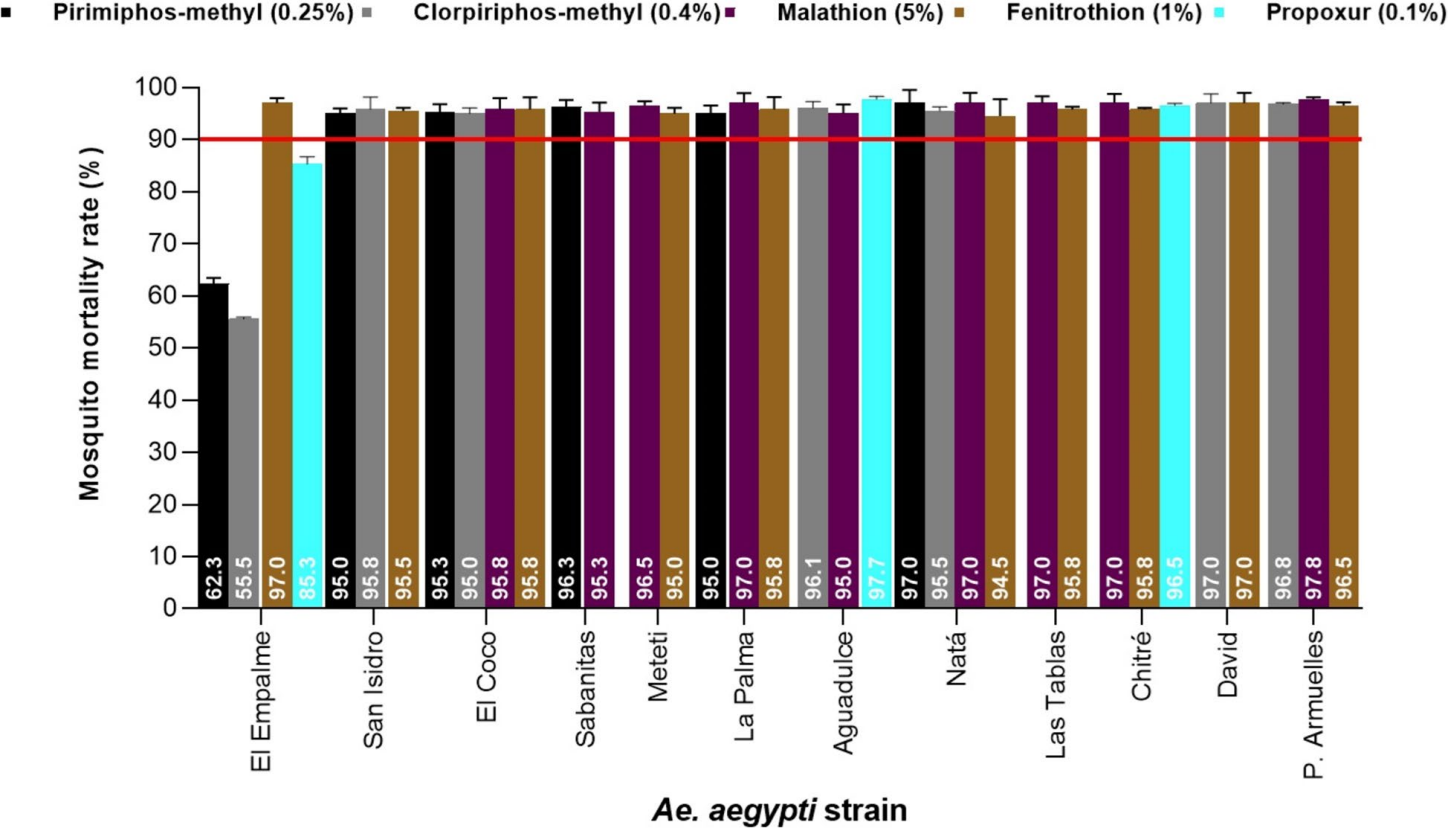
Resistance status in populations of *Ae. aegypti* adults evaluated by diagnostic concentrations of organophosphates and carbamate insecticides using WHO standardized bioassays. The continuous red line shows the threshold value of 90%, below this value a population is considered resistant to insecticides

Bioassays with OPs insecticides showed a higher number of *Ae. aegypti* strains with possible resistance, and only *Ae. aegypti* El Empalme showed confirmed resistance to pirimiphos-methyl (62.3%), chlorpyrifos-methyl (55.5%) and propoxur (85.3%). *Aedes aegypti* strains 24 Diciembre and Ustupo failed to register any resistance to OPs and CA insecticides. Figure 3 shows the resistance status of the different *Ae. aegypti* populations to OPs insecticides and CA propoxur.

**Fig. 4:**
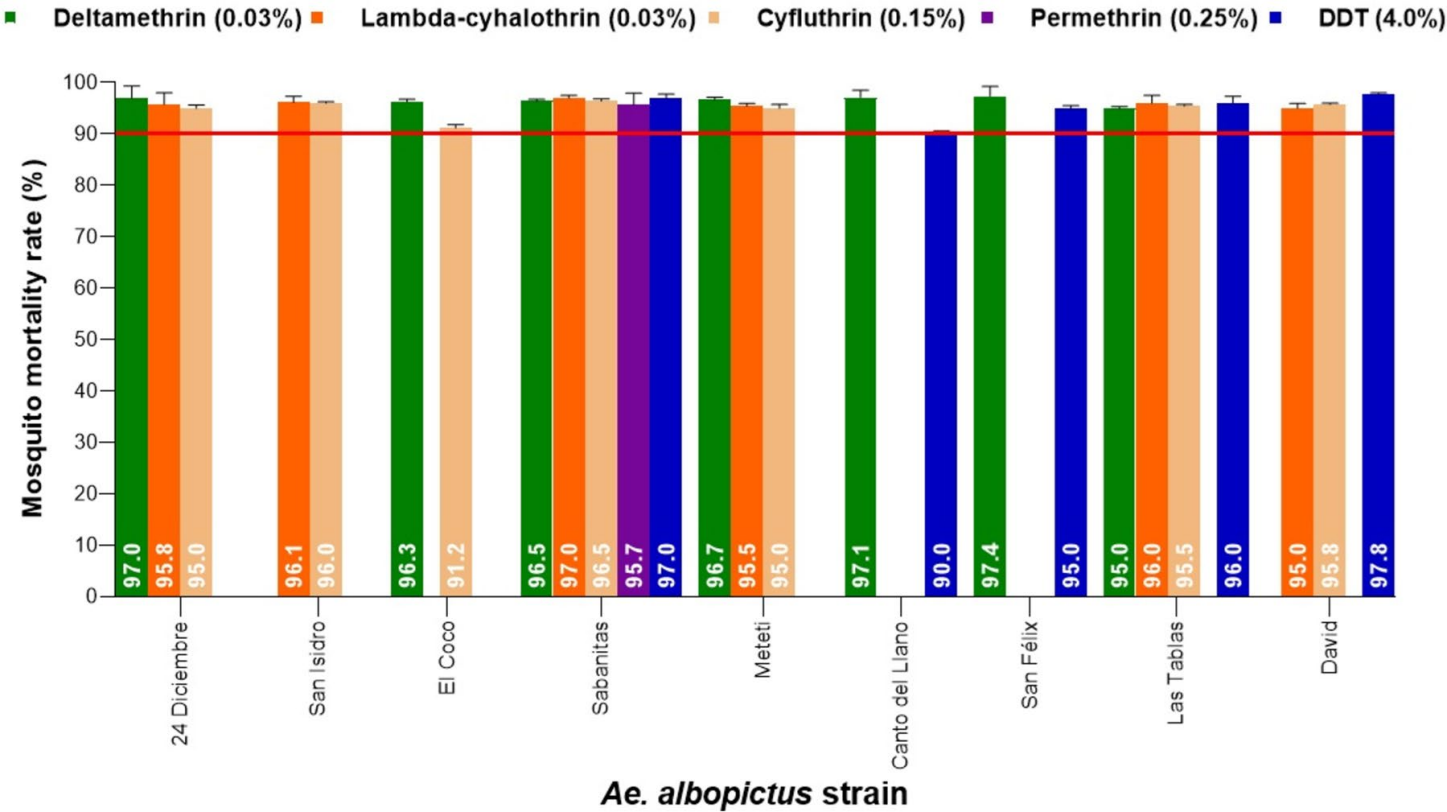
Resistance status in populations of *Ae. albopictus* adults evaluated by diagnostic concentrations of pyrethroids and organochlorine insecticides using WHO standardized bioassays. The continuous red line shows the threshold value of 90%, below this value a population is considered resistant to insecticides

**Fig. 5:**
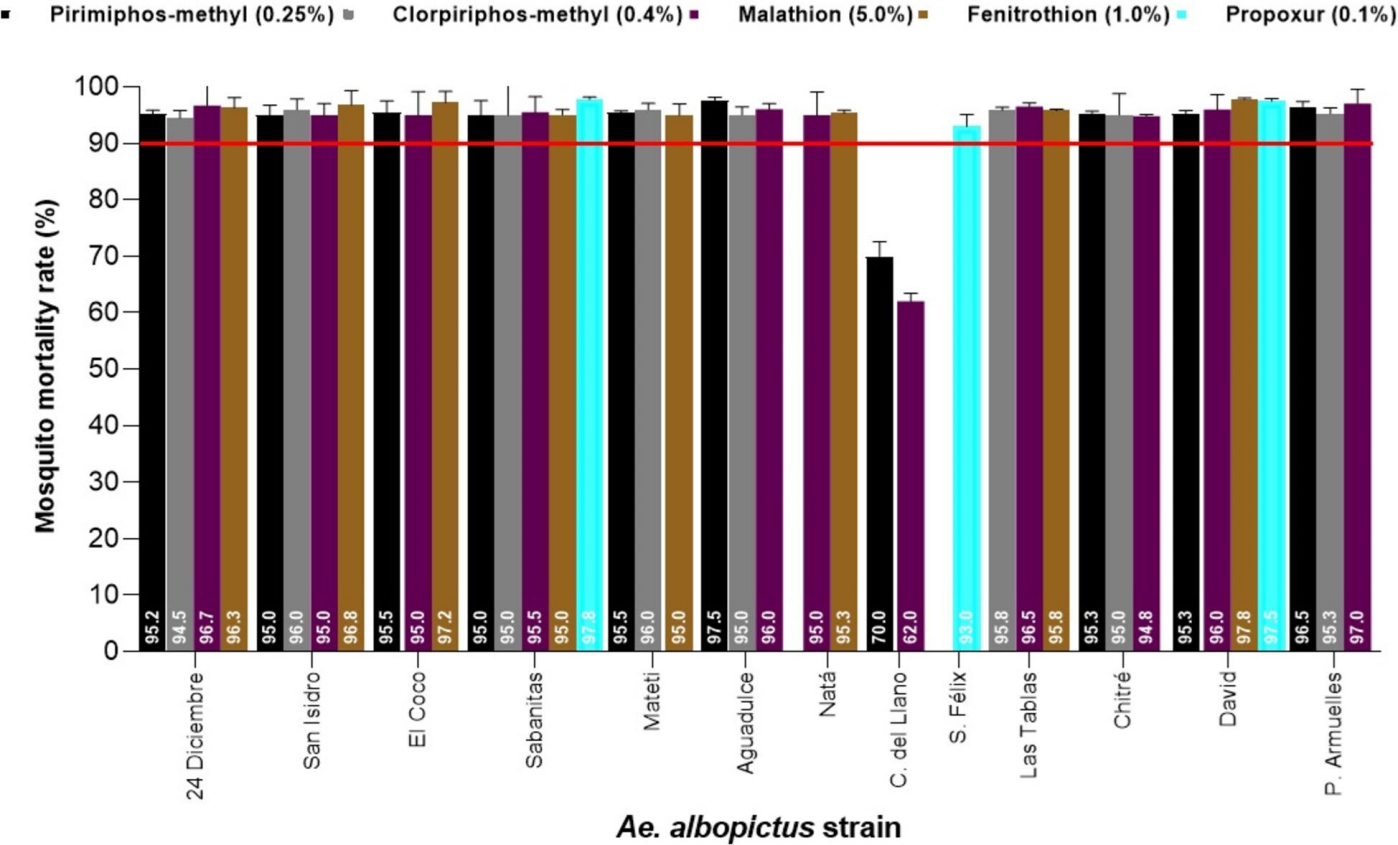
Resistance status in populations of *Ae. albopictus* adults evaluated by diagnostic concentrations of organophosphates and carbamate insecticides using WHO standardized bioassays. The continuous red line shows the threshold value of 90%, below this value a population is considered resistant to insecticides

*Aedes albopictus* was collected in 13 urbans communities and semi-urbans; only nine strains of *Ae. albopictus* showed possible resistance to the PYs insecticides evaluated, while the strains of *Ae. albopictus* Sabanitas, Canto del Llano, San Félix, Las Tablas and David showed possible resistance to DDT. It should be noted that *Ae. albopictus* Sabanitas showed possible resistance to all the PYs evaluated and to DDT; resistance to DDT may be due to a possible cross-resistance with deltamethrin. Figure 4 shows the resistance status of the different populations of *Ae. albopictus* to PYs insecticides and to the organochlorine DDT. With OPs insecticides, 13 strains of *Ae. albopictus* showed possible resistance, and only *Ae. albopictus* Canto del Llano showed confirmed resistance to pirimiphos-methyl (70%) and malathion (62%), while *Ae. albopictus* Sabanitas, San Félix and David showed possible resistance to propoxur. Figure 5 shows the resistance status of the different *Ae. albopictus* populations to OPs insecticides and CA propoxur. The percentage of mortality registered in the controls in the field strains of *Ae. aegypti* and *Ae. albopictus* was less than 4%.

### Biochemical assays

#### α- and β-Esterases

**Fig. 6:**
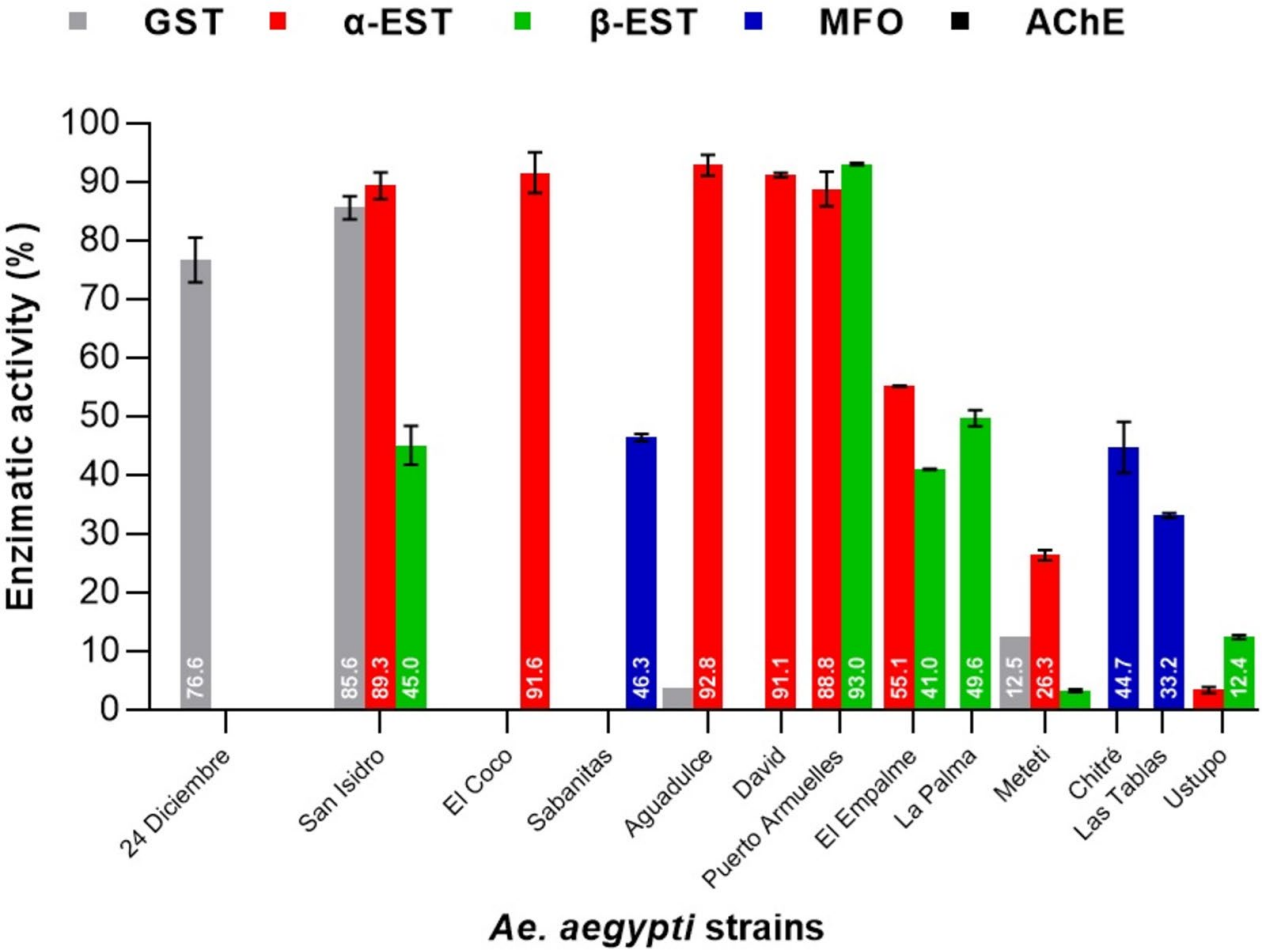
Enzyme activity levels in field populations of *Ae. aegypti* compared to the reference strain *Ae. aegypti* Rockefeller susceptible to insecticides. Enzyme activity was classified as “unaltered” between 0 and 15%, “incipient altered” between 15 and 50% and “altered” between 50 and 100% [43]

**Fig. 7:**
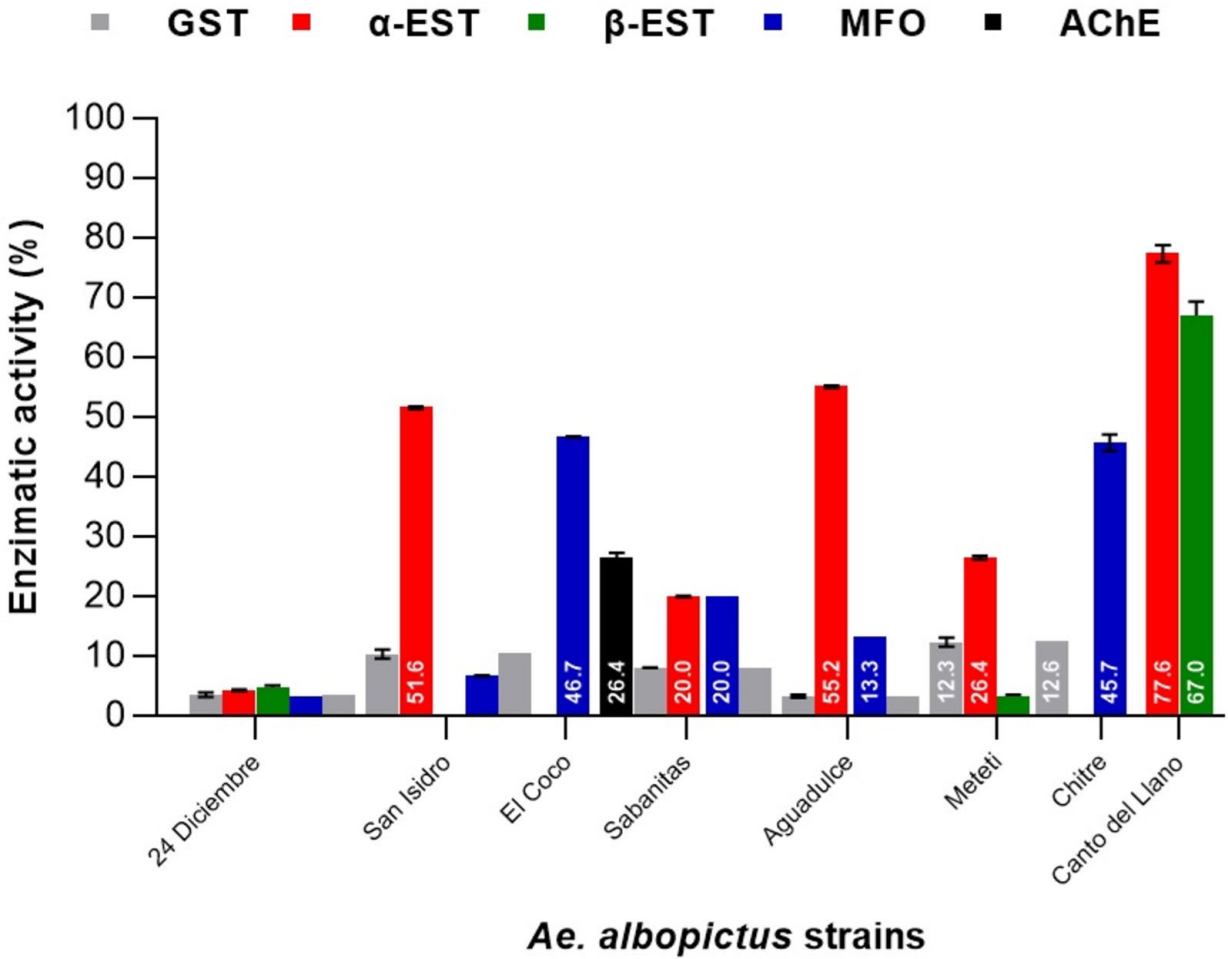
Enzyme activity levels in field populations of *Ae. albopictus* compared to the reference strain *Ae. aegypti* Rockefeller susceptible to insecticides. Enzyme activity was classified as “unaltered” between 0 and 15%, “incipient altered” between 15 and 50% and “altered” between 50 and 100% [43]

Esterase activity was variable across sites with the most frequent detection of α-EST compared to β-EST in *Ae. aegypti* populations. The strains *Ae. aegypti* San Isidro (89.3%), *Ae. aegypti* El Coco (91.6%), *Ae. aegypti* Aguadulce (92.8%), *Ae. aegypti* David (91.1%), Puerto Armuelles (88.8%) and *Ae. aegypti* El Empalme (55.1%) showed altered activity level of α-EST, while altered activity level of β-EST was only observed in the *Ae. aegypti* Puerto Armuelles (93.0%). The strains *Ae. aegypti* San Isidro (45%), *Ae. aegypti* El Empalme (41%) and *Ae. aegypti* La Palma (49.6%) showed incipient activity level of β-EST (Fig. 6). *Aedes albopictus* only showed altered activity level of α-EST in *Ae. albopictus* San Isidro (51.6%), *Ae. albopictus* Aguadulce (55.2%) and *Ae. albopictus* Canto del Llano (77.6%). The strains *Ae. albopictus* Meteti (26.4%) and *Ae. albopictus* Sabanitas (20%) showed incipient activity level of α-EST. Only the strain *Ae. albopictus* Canto del Llano (67%) showed altered activity levels of β-EST (Fig. 7). Overall, *Ae. aegypti* and *Ae. albopictus* populations showed a higher degree of both α-EST and β-EST activity compared to the susceptible Rock strain, suggesting the presence of this OPs insecticide resistance mechanism.

#### Mixed function oxidases

Determination of MFOs activity is an important factor in addressing metabolic resistance studies. The *Ae. aegypti* Sabanitas (46.3%), *Ae. aegypti* strains Chitré (44.7%) and *Ae. aegypti* Las Tablas (33.2%) were the only strains that showed incipient activity level of MFO (Fig. 6). *Aedes albopictus* El Coco (46.7%), *Ae. albopictus* Chitre (45.7%) and *Ae. albopictus* Sabanitas (20%) showed incipient activity level of MFOs. (Fig. 7).

#### Glutathione-*S*-transferase

Altered GST activity was only detected in *Ae. aegypti* 24 Diciembre (76.6%) and San Isidro (85.6%). This activity may be associated with the possible resistance recorded in this mosquito strains to DDT. This possible resistance to DDT may be due to the presence of cross-resistance with PYs insecticides (Fig. 6). No GST enzymatic activity was detected in the *Ae. albopictus* strains (Fig. 7).

#### Acetylcholinesterase

Acetylcholinesterase activity was not detected in any of the *Ae. aegypti* strains (Fig. 6). Incipient activity level of AchE was recorded only in *Ae. albopictus* Sabanitas of 26.4% (Fig. 7).

Through ANOVA, significant differences were established in the levels of enzymatic activity of α- and β-EST, MFOs and GST, with *p* < 0.001 in the *Ae. aegypti* and *Ae. albopictus.* Using the Tukey test, significant differences were also shown in the enzyme levels between strains of *Ae. aegypti* and *Ae. Albopictus* with *p* < 0.001. Alpha-esterase showed significant differences between most strains, except for the α-EST that did not show significant differences between the *Ae. aegypti* El Coco and Aguadulce, between *Ae. aegypti* El Coco and David, and between *Ae. aegypti* San Isidro and David.

#### Detection of Val1016Ile and F1534C sodium channel mutations

A total of 62 mosquitoes corresponding to *Ae. aegypti* Aguadulce, El Coco and David were examined for detection and identification of Val1016Ile and F1534C sodium channel mutations. We detected the Val1016Ile mutation in the VGSC gene. The F1534C mutation was not detected. In general, the presence of the *kdr* mutation was observed in the three populations evaluated, and the results showed that the total frequency was significantly higher in the resistant population *Ae. aegypti* El Coco with 94.4%, a mean of 63.3%, median of 59.1% and range of 36.4%–94.4% for the three populations (*X*^2−test^, *p* < 0.05; Table 3). The odds ratio for the Val1016Ile mutation ranged from 0.8 to 20.8 in resistant mosquitoes, indicating the association between pyrethroid phenotypic resistance and the *kdr* mutation (Table 3). The frequency of the homozygous wild genotype (V/V) was significant, and a higher frequency was observed in *Ae. aegypti* David (Fg = 47.4%). The homozygous mutant genotype (I/I) was significant with a higher frequency in *Ae. aegypti* Aguadulce (Fg = 54.6) and the heterozygous genotype (V/I) was also significant, showing a higher frequency *Ae. aegypti* El Coco (Fg = 42.9%) (Table 4). All three genotypes at position 1016 were represented. This study makes the first records of the Val1016Ile mutation in these studied sites.

**Table 3:** *Kdr* mutation frequency relationship to pyrethroid resistance in different *Aedes aegypti* populations

*Ae. aegypti* populations	Phenotype	Sample Size	Allele Frequency %		Odds ratio (95% CI)
			Wildtype	Val1016Ile	Val1016Ile
David	R	22	14 (63.6)	8 (36.4)	0.8 (0.2–2.9)^NS^
Aguadulce	R	22	9 (40.9)	13 (59.1)	NA
El Coco	R	18	1 (5.6)	17 (94.4)	20.8 (3.3–411.9)^***^

*NS* no significance, *NA* not applicable

^***^ *p* < 0.001

**Table 4:** Genotyping for 1016 result

*Ae. aegypti* Strains	*N*	Genotype frequency (%)			*X*^2^	d*f*	*p*-value
		1016 (V/V)	1016 (V/I)	1016 (I/I)			
David	19	47.4%	31.6%	21.1%	12.3	4	0.015
Aguadulce	22	13.6%	54.6%	31.8%			
El Coco	21	4.8%	52.4%	42.9%			

*N*, Sample size; Genotype frequency, Wild Homozygous (V/V), Heterozygous (V/I) and Mutant Homozygous (I/I). Statistical values: *X*^2^: Chi-square, d*f*: degree of freedom, *p*-value: significant differences (*p* < 0.05)

## Discussion

### Susceptibility bioassays

The heterogeneity of insecticide resistance patterns shown by *Aedes* populations suggests that several mechanisms may be contributing to the development of resistance. Despite the use of PYs insecticides for the past 25 years, little work has been done to determine the state of the susceptibility in *Aedes* populations in the main regions of dengue transmission in Panama. The resistance detected to deltamethrin and lambda-cyhalothrin in *Ae. aegypti* El Coco, David and Aguadulce, respectively, represents a technical problem for the PNCA. Previous studies detected possible resistance in *Ae. aegypti* Puerto Armuelles to deltamethrin, lambda-cyhalothrin and cyfluthrin, *Ae. aegypti* David to deltamethrin and lambda-cyhalothrin, *Ae. aegypti* Aguadulce and El Empalme to lambda-cyhalothrin and cyfluthrin, *Ae. aegypti* 24 Diciembre to cypermethrin, cyfluthrin and chlorpyrifos-methyl and DDT [26, 28]. The resistance of *Ae. aegypti* to PY has been reported in several countries in South America, Central America and North America; some African countries, several in Asia and Oceania [13]. In the Americas, deltamethrin and permethrin resistance has been reported in *Ae. aegypti* in Mexico [44], in El Salvador to deltamethrin and cypermethrin [45], in Costa Rica to cypermethrin [46], in Colombia to lambda-cyhalothrin and permethrin [47], in Cuba to lambda-cyhalothrin and deltamethrin [48, 49] and in Brazil to deltamethrin is found in almost all states [50, 51]. Although the PNCA only uses deltamethrin and cyfluthrin, the recorded possible resistance to lambda-cyhalothrin and permethrin may be due to the presence of cross-resistance. The resistance detected to DDT in the strains of *Ae. aegypti* 24 Diciembre (60.2% mortality) and *Ae. aegypti* San Isidro (75.7% mortality) may also be due to cross-resistance with deltamethrin. The manifestation of cross-resistance conferred to pyrethroids limits the number of suitable alternatives for vector control [52]. Previous work has detected cross-resistance of deltamethrin with permethrin in *Ae. aegypti* [53, 54], as well as of lambda-cyhalothrin with permethrin [55]. Resistance may also be due to the intensified use of domestic commercial insecticides, those used by pest control companies and public health activities during each new outbreak [56–58].

*Aedes albopictus* Canto del Llano only registered resistance to pirimiphos-methyl (70% mortality) and malathion (62% mortality). Studies conducted in Mexico with *Ae. albopictus* showed similar results with resistance to chlorpyrifos-methyl, malathion, permethrin and deltamethrin [44]. On the other hand, in a study conducted in Cuba, resistance to temephos and deltamethrin was detected in *Ae. albopictus* Mulgoba, while *Ae. albopictus* Plaza was observed only resistance to lambda-cyhalothrin [59]. A separate study indicated that *Ae. albopictus* rapidly generated high resistance to the most used insecticides for adult mosquitoes (deltamethrin and permethrin) and to the larvicide temephos [60]. A study carried out in Pakistan indicated that the resistance detected in *Ae. albopictus* could be related to its presence in crop areas and its indirect exposure to different agrochemicals [61]. It can be assumed that resistance to malathion may be due to cross-resistance with pirimiphos-methyl since this insecticide is frequently used by pest control companies. Other study showed in *Ae. albopictus* in Malaysia cross-resistance between four organophosphates, as well as cross-resistance between one organochlorine and two organophosphates [62]. On the other hand, the susceptibility or resistance status of temephos against *Ae. albopictus* may vary due to significant differences in the weekly levels of EST, MFOs, GST, and insensitive AchE [63]. In Panama, little is known about the susceptibility of *Ae. albopictus* to insecticides and the influence of coexistence with *Ae. aegypti*.

In general, all *Ae. aegypti* and *Ae. albopictus* populations showed varied behavior of resistance status to the insecticides evaluated, resistance levels varied among study sites and with different insecticides. This may be due to differences in insecticide use at the local level. This may suggest that variable susceptibility behavior may be because the underlying mechanisms causing resistant phenotypes in these populations may not be shared [64]. Therefore, the resistance phenotype can be generated by a large number of different mechanisms that result in mosquito populations that differ in the spectrum and level of resistance to different insecticides. Furthermore, resistance can vary temporally and spatially between insecticides and mosquito species within and outside the country [65]. Consequently, the mechanisms of resistance need to be further investigated. Likewise, enzymatic mechanisms present at the study sites need to be considered [66].

### Biochemical assays

#### Esterases

Evaluation of metabolic resistance was achieved with biochemical tests quantifying the activity of the main classes of detoxifying enzymes. The esterases are strongly associated with resistance in mosquitoes to OPs, CA and to a lesser extent PYs insecticides [66–69]. The increased activity of esterases in *Ae. aegypti* and *Ae. albopictus* populations especially α-EST suggests an important role of this enzyme in the metabolic mechanisms conferring resistance to the insecticides evaluated. In *Ae. aegypti*, altered activity levels of α-EST were detected in six populations (*Ae. aegypti* San Isidro, El Coco, Aguadulce, David, Puerto Armuelles and El Empalme), and in three strains, it was associated with confirmed resistance to deltamethrin and lambda-cyhalothrin (*Ae. aegypti* Aguadulce, El Coco and David). The altered activity levels of α-EST may also be due to the resistance detected to DDT in the strain *Ae. aegypti* San Isidro as a result of cross-resistance with deltamethrin. In addition, this altered activity level of α-EST may probably be involved in the possible resistance detected mainly to PYs in the studied localities, and this needs to be confirmed through enzymatic studies. Only *Ae. albopictus* San Isidro (51.6%), Aguadulce (55.2%) and Canto del Llano (77.6%) recorded altered activity level of α-EST. Altered activity levels of β-EST were detected only in *Ae. aegypti* Puerto Armuelles (93%) and *Ae. aegypti* San Isidro (45%), El Empalme (41%) and La Palma (49.6%) recorded incipient altered activity level and *Ae. albopictus* Canto del Llano recorded altered activity level of β-EST. In studies conducted in Colombia with *Ae. aegypti*, incipient activity of α-EST was detected where resistance profiles were highly correlated with permethrin and lambda-cyhalothrin followed by deltamethrin and cyfluthrin, showing susceptibility to the OPs tested [70]. Studies to date have reported overexpression of β-EST in OP- and PY-resistant populations [71–73]. In more recent study, highly altered activity of α-EST and β-EST was detected in one population and incipient activity in three *Ae. aegypti* populations [47]. In a study conducted with *Ae. aegypti*, EST and GST were found to be among the most strongly involved metabolic resistance mechanisms throughout Brazil. They were also closely correlated with resistance to OPs and PYs in Brazilian samples [74].

#### Mixed function oxidases

In this study, *Ae. aegypti* Sabanitas (46.3%), Chitre (44.7%) and Las Tablas (33.2%) showed incipient activity level of MFOs, and also *Ae. albopictus* El Coco (46.7%) and Chitre (45.7%) showed incipient activity level. These levels of incipient activity of MFOs may be associated with the possible resistance detected to PYs. It can be said from the results obtained in this study that metabolic mechanisms play an important and potential role in the development of resistance in *Ae. aegypti* and *Ae. albopictus* populations and, among these mechanisms, MFOs. Mixed function oxidases are often implicated in resistance to PYs and, to a lesser extent, to OPs insecticides [34, 47]. On the other hand, MFOs have been described as important in the detection of resistance to OPs insecticides in Latin American samples [34, 75]. Most studies conducted with *Ae. aegypti* from different regions of Colombia have detected altered activity mainly of MFOs and EST, associating this activity with resistance to PYs [47]. In other countries, alterations of α-EST, β-EST and MFOs have been reported in *Ae. aegypti* resistant to organophosphates, carbamates and pyrethroids [76–78]. In a study conducted in Brazil, changes in MFO activity were less prominent, with only about 30% and less than 20% of adult and larval samples, respectively, exhibiting changes in MFO activity [74]. This heterogeneity of resistance patterns within the pyrethroid class suggests that diverse mechanisms are contributing to these phenotypes.

#### Glutathione *S*-transferase

This enzyme system is generally involved in insect resistance to OPs insecticides and provides the most important form of metabolic resistance to DDT through dehydrochlorination to DDE and pyrethroid resistance [79, 80]. In this study, the DDT confirmed resistance detected in the *Ae. aegypti* 24 Diciembre (60.2% mortality) and San Isidro (75.7% mortality), and this resistance may be associated with the alteration of the level of GST altered activity detected in strains *Ae. aegypti* 24 de Diciembre (76.6%) and San Isidro (85.6%), respectively. On the other hand, the low levels of GST activity detected in *Ae. aegypti* and *Ae. albopictus*, can be interpreted as an initial enzymatic activity that should be followed up in future studies to determine its involvement in the development of resistance to OC and/or PYs insecticides. In Colombia, the GST-based mechanism was associated with DDT resistance in *Ae. aegypti* [70] and it was suggested that it may also play a role in resistance to PYs [70, 81]. The activity of α-EST and GST is an important mechanism in PYs resistance in *Ae. aegypti* [82]. On the other hand, GST activity is associated with resistance to permethrin and deltamethrin [64]. The detection of altered ESTs and GST activity seen in our study suggests the presence of multiple resistance mechanisms involved in *Ae. aegypti* populations. Although DDT was used from 1962 until almost the end of the 1980s for malaria eradication, its use was stopped in 1988 [24]. Few studies have shown whether the DDT-resistant phenotype may still be present in *Ae. aegypti* [83]. Furthermore, DDT and pyrethroids share the same mode of action on voltage-dependent sodium channels and the observed resistance may be due to the extensive use of pyrethroids in pest control and public health activities. There is the possibility of the occurrence of cross-resistance between PYs and DDT [57].

#### Acetylcholinesterase

Structural changes at this site have resulted in the development of resistance in many insect vectors [84]. Mutations in the AchE enzyme can prevent OPs binding to the active sites, thus decreasing or eliminating the efficacy of these insecticides [85]. In this study, in the 13 strains of *Ae. aegypti* evaluated by biochemical tests, no AchE’s altered activity levels were detected. The *Ae. albopictus* Sabanitas population was the only one that showed differences in medians with statistical significance compared to the *Ae. aegypti* Rock strain (*p* < 0.05), with record of incipient AchE activity (> 15% and < 50%). This suggests that the target site is still sensitive, and that this enzyme does not show so far represents an important enzymatic mechanism of resistance to OPs and CA insecticides. However, further studies are needed to confirm this hypothesis. In a previous study with *Ae. aegypti* 24 Diciembre, no altered AchE activity was detected [28], confirming with this study a similar enzymatic behavior in this mosquito strain. In studies conducted in Brazil, similar values of AchE (> 15% and < 50%) were observed in three populations of *Ae. aegypti*, only *Ae. aegypti* Mossoró strain presenting resistance to OPs temephos, which was associated with altered AchE activity [86]. In comparison, with another work with *Ae. aegypti* Rio, no altered AchE activity was detected [87]. In a similar study, very high AchE activity was found in all *Ae. aegypti* field populations and it was suggested that this represented another potential mechanism for resistance to OPs insecticides [88]. In Bangladesh, two *Ae. aegypti* populations were detected with elevated levels of AchE that was associated with malathion resistance [64]. In general, there are few studies on *Aedes* populations where altered AchE activity and its association as a mechanism of insecticide resistance have been detected in *Aedes* populations. Considering that this study represents the first report of the profile of the enzymes associated with insecticide resistance mechanisms in *Ae. aegypti* and *Ae. albopictus* populations, it is necessary that more studies like this are carried out in future.

#### Genotyping of *Kdr* mutations

In this study, we report for the first time the detection of the Val1016Ile mutation in three strains of *Ae. aegypti* (El Coco, Aguadulce and David) and provide strong evidence that this mutation may be contributing to the PYs resistance deltamethrin and lambda-cyhalothrin. In the only previous study conducted on the *Kdr* gene, the Val1016Gly mutation was detected in an *Ae. aegypti* population from central Panama [29]. These results show a high frequency of homozygous mutant (Ile/Ile) genotype in Panamanian *Ae. aegypti* population from Aguadulce and El Coco. This finding is consistent with those reported by other studies that obtained similar results in Caribbean *Ae. aegypti* populations [36]. The intensive use of insecticides exerts a strong selection pressure on mosquitoes, favoring the increase of resistance alleles in natural populations. For example, in Mexico, after 6 to 8 years of excessive permethrin use, a significant increase in a mutation at position AaNaV 1016 was observed, reducing the efficacy of chemical control over time [89]. There are two mutations described in Latin America, the Val1016Gly and Val1016Ile allele, respectively [19, 36, 90] and in Southeast Asia [91]. The Val1016IIe mutation associated with PYs resistance in *Ae. aegypti* has been repeatedly detected in resistant populations in the Americas. The Val1016Ile allele has been detected in *Ae. aegypti* populations in Mexico associated with resistance to permethrin and deltamethrin [92], in Brazil to PYs insecticides [90], in Cuba to deltamethrin [93], in Peru to PYs [38], and in Colombia, it was associated with resistance to lambda-cyhalothrin [94]. It is important to characterize these mutations before new adaptive alleles can be selected to lessen the negative effects of the *Kdr* gene [95]. It is possible that there are regional differences in *Ae. aegypti* collection sites related to *Kdr* mutations [96]; however, this hypothesis requires further research work. In this study, it was not possible to test all populations of *Ae. aegypti* and *Ae. albopictus*. This study shows F1534C mutation is absent in *Ae. aegypti* populations studied. However, several studies report how F1534C mutation has been detected in pyrethroid-resistant *Ae. aegypti* populations from Cuba [93], Grand Cayman [36] Mexico [97–99], Venezuela [21], Brazil [100], Puerto Rico [101], Colombia [22], China [96], Vietnam [102], Laos [103], Peru [38], India [104], Portugal [105], Saudi Arabia [106], and Burkina Faso [107].

This study serves to establish a baseline on the state of resistance in *Ae. aegypti* and *Ae. albopictus*, and further studies should be carried out to see how the behavior of resistance and/or susceptibility of these mosquito populations develops in different sites of epidemiological and entomological importance. The relevance of these results from a technical/operational perspective is that they will substantially guide the PNCA in the selection of effective alternative insecticides for the control of *Aedes* populations. As for the detection of confirmed resistance to the pyrethroids deltamethrin and lambda-cyhalothrin, it requires the replacement of these insecticides by alternative insecticides, previously evaluated for their effectiveness. The use of insecticides with synergistic agents such as PBO may be an alternative to mitigate the negative effects of resistance caused by the pyrethroid insecticides applied. Finally, based on the results of this study and previous works [21–25], we can indicate that insecticide resistance in the populations mainly of *Ae. aegypti* and *Ae. albopictus* has increased in recent years in the different endemic regions of the country. This raises several implications that should be considered by the NACP: (1) the selection of insecticides should be made based on previous susceptibility and/or resistance studies that indicate the efficacy and effectiveness of the insecticide molecule to be used, (2) within the vector control strategies carried out at the level of the health regions, the surveillance and management of insecticide resistance should be highlighted, (3) the application of insecticides should be based on a strategy of appropriate use of insecticides to reduce the risks of the resistance development, slow down its evolution or reverse it to a level compatible with the efficient use of insecticides for vector control, and (4) Vector control management should be carried out with the support of scientific evidence to increase the effectiveness of interventions against disease-transmitting mosquito populations.

## Limitations

One of the limitations of the study was that it was not possible to detect allelic variants of the *Kdr* gene in all the *Ae. aegypti* populations under study, and the same analysis could not be performed with the *Ae. albopictus* populations. However, the three *Ae. aegypti* populations evaluated were very similar in terms of environmental, ecological and operational aspects to the other populations located in the same regions studied.

## Conclusion

The presence of a varied and generalized resistance to insecticides, the Val1016IIe mutation in *Ae. aegypti* and *Ae. albopictus* in different geographic regions may be associated with the intensive use and possibly misuse of the different insecticides applied to control *Aedes* populations. It is necessary to develop a program for monitoring, surveillance and resistance management. Also, alternative approaches to mosquito control that do not involve insecticides should be explored.

## Data Availability

The data sets analyzed during the current study are available from the corresponding author on reasonable request.

## Abbreviations

Ache: Acetylcholinesterase
CA: Carbamate
CCE: Carboxylesterases
CHIKV: Chikungunya virus
CL50: Lethal concentrations 50%
CYPs: Cytochrome P450 Monooxygenases
DDT: Dichlorodiphenyltrichloroethane
DE: Epidemiological Department
DENV: Virus dengue
DME: Department of Medical Entomology
EST: Esterase
RR: Resistance ratio
GST: Glutathione *S*-transferases
ICGES: Instituto Conmemorativo Gorgas de Estudios de la Salud
LC: Lethal concentrations
MFOs: Mixed function oxidases
MINSA: Ministerio de Salud
NaV: Voltage-gated sodium channel
NSEs: Nonspecific esterases
OC: Organochlorines
OPs: Organophosphates
PAHO: Pan American Health Organization
PBO: Piperonyl butoxide
PCR: Polymerase chain reaction
PNCA: Programa Nacional de Control de *Aedes*
PYs: Pyrethroids
Rock: Rockefeller
SENACYT: National Secretariat of Science, Technology and Innovation
SIN: National Research System
ULV: Ultra-low volume
WHO: World Health Organization
ZIKV: Zika virus
